# Glutathione Delivery Using a Furcellaran–Chitosan System: Effects of Microencapsulation and Incorporation Strategy on Digestion in a Food Matrix

**DOI:** 10.3390/ijms27093803

**Published:** 2026-04-24

**Authors:** Joanna Tkaczewska, Wiktoria Grzebieniarz, Małgorzata Morawska-Tota, Krzysztof Szostak, Beata Synkiewicz-Musialska, Ewelina Jamróz

**Affiliations:** 1 Department of Animal Products Processing, Faculty of Food Technology, University of Agriculture in Kraków, Balicka 122, 30-149 Kraków, Poland; 2 Department of Chemistry, Faculty of Food Technology, University of Agriculture in Kraków, Balicka 122, 30-149 Kraków, Poland; wiktoria.grzebieniarz@urk.edu.pl (W.G.); ewelina.jamroz@urk.edu.pl (E.J.); 3 Department of Sports Medicine & Human Nutrition, Faculty of Physical Education and Sport, University of Physical Culture in Krakow, al. Jana Pawła II 78, 31-537 Kraków, Poland; malgorzata.morawska@awf.krakow.pl; 4 Łukasiewicz Research Network—Institute of Microelectronics and Photonics, Kraków Division, Zabłocie 39, 30-701 Kraków, Poland; krzysztof.szostak@imif.lukasiewicz.gov.pl (K.S.); beata.synkiewicz.musialska@imif.lukasiewicz.gov.pl (B.S.-M.); 5 Department of Product Packaging, Faculty of Commodity Science and Product Management, Cracow University of Economics, 27 Rakowicka St., 31-510 Kraków, Poland

**Keywords:** capsules, glutathione, furcellaran, food matrix, in vitro digestion, functional food

## Abstract

The aim of this study was to evaluate the impact of glutathione (GSH), applied in free form and in the form of microcapsules, on the release and nutritional properties of a functional post-exercise recovery snack. Five snack variants enriched with free GSH or microencapsulated GSH, applied using different incorporation strategies, were prepared and subjected to standardised in vitro digestion following the INFOGEST protocol. The study assessed GSH levels in the digesta, protein and fat digestibility, antioxidant capacity, and changes in fatty acid profiles after digestion. Snacks fortified with free GSH exhibited the highest immediate GSH levels in the digesta (up to 2390 ± 240 nmol/mL), whereas lower levels were observed for microencapsulated GSH (down to 1280 ± 132 nmol/mL), reflecting differences in release behaviour under in vitro digestion conditions. Products containing microencapsulated GSH showed a markedly higher post-digestion free amino acid content (1520 ± 100 mg/100 g) compared with those enriched with free GSH (820.3 ± 19 mg/100 g), indicating differences in the profile of protein digestion products. Antioxidant activity and phenolic content increased after digestion across all formulations, with no consistent differences between variants, while fat digestibility remained unchanged, although fatty acid profiles differed depending on the GSH application form. Overall, the results indicate that the form of GSH incorporation, including microencapsulation, influences its behaviour during in vitro digestion and affects the release and distribution of compounds within the food matrix.

## 1. Introduction

More and more people are beginning to recognise the importance of physical activity in maintaining health. As the intensity of physical activity increases, the body’s demand for oxygen also rises, which leads to increased respiratory processes and production of reactive oxygen species [1]. Reactive oxygen species, although often associated with oxidative damage, also play a vital role in physiological adaptation to exercise, including muscle regeneration and regulation of transcription factors involved in cellular signalling. However, excessive amounts of free radicals can contribute to oxidative stress, which negatively affect muscle function and post-exercise regeneration. Therefore, supporting the body with appropriate nutritional and supplementation strategies is becoming a key element of modern sports. The use of specialist methods and means is not only aimed at accelerating post-exercise regeneration, but also at protecting the joints and replenishing the loss of energy, micro- and macro-elements necessary to maintain the body’s efficacy [2]. The dietary supplement market is dynamically developing, encompassing not only people who train professionally, but also the recreationally active population. Although the range of supplements for athletes is constantly expanding, only some of them have proven effectiveness [2]. One promising ingredient with the potential to support the body of athletes is glutathione (GSH)—a tripeptide naturally synthesised by the human body and plants. This compound is a very strong hydrophilic antioxidant that affects the maintenance of redox balance, reduction in oxidative stress, enhancement of metabolic detoxification and regulation of immune system functions. The difficulty with using it as an oral supplement is its high susceptibility to chemical and enzymatic decomposition by digestive enzymes. Hydrolysis of glutathione GSH by γ-glutamyltransferase results in poor absorption from the gastrointestinal tract [3]. Encapsulating GSH in a protective multilayer material may reduce its susceptibility to enzymatic and chemical degradation during gastrointestinal transport. This approach holds promise for increasing the bioaccessibility and efficiency of orally administered glutathione supplements, particularly in the context of potential therapeutic applications.

Multilayer microcapsules based on furcellaran (FUR) and chitosan (CHIT) have been previously developed as carriers for glutathione delivery. Earlier studies demonstrated that these systems can protect the encapsulated compound against environmental and gastrointestinal degradation and exhibit biological activity under specific experimental conditions [4,5]. In particular, in vivo studies have indicated that oral administration of encapsulated glutathione may influence its systemic availability and antioxidant-related responses. However, such findings should be interpreted with caution in the context of simplified in vitro digestion models, as the underlying mechanisms and conditions differ substantially. Therefore, further investigation under controlled in vitro conditions is required to better understand the behaviour of glutathione incorporated into food matrices, especially with respect to its bioaccessibility and interactions with other food components during digestion.

In vitro studies on digestion processes are one of the key methods used in the development and validation of microencapsulation systems for bioactive compounds, enabling the assessment of their stability and bioaccessibility in conditions simulating the gastrointestinal environment. The properties of an effective microcapsule should not only protect the active substance and increase its bioaccessibility, but also enable its controlled release in specific conditions of the gastrointestinal tract. In many studies, it has been shown that microencapsulation of various bioactive ingredients improves their stability and increases bioaccessibility after simulated in vitro digestion [6]. However, the majority of studies are focused on analysing the digestion of the microencapsulated substance itself, without considering its effects on the digestion of other nutrients in the complex food matrix. There is a lack of research on how microcapsules with an active ingredient affect the digestion of proteins and lipids in a complex food product for special nutritional purposes, which is a significant research gap in the context of functional foods.

An important element of this research was the development of an innovative snack supplemented with glutathione, intended for physically active people and adapted to the needs of peri-exercise nutrition. However, the key objective was to implement an innovative system of microencapsulation of glutathione and to assess its bioaccessibility from the food matrix after in vitro digestion. The influence of microcapsules on the digestibility of other matrix components and the properties of the entire product after digestion was also analysed, with particular attention paid to different methods of glutathione application. The effectiveness of adding glutathione to the interior of the product and applying it to its surface using electrospray was compared to determine which method provides better protection against enzymatic degradation. The in vitro analyses allowed us to assess the bioaccessibility of glutathione and its effects on the digestion of proteins and lipids in the food matrix, emphasising the innovative nature of the applied technologies.

## 2. Results and Discussion

Bilayer microcapsules based on the Furcellaran (FUR)—Chitosan (CHIT) system, enriched with glutathione (GSH), were obtained by Drozdowska, Piasna-Słupecka [4]. A key result of the study was the demonstration of a relationship between cell growth inhibition and the ability of GSH, both free and incorporated into FUR/CHIT structures, to induce apoptosis in cancer cell lines. The obtained results indicate that the tested capsules led to cell elimination primarily through the activation of apoptotic mechanisms. Furthermore, data analysis suggests that the observed reduction in proliferation and induction of cell death in the G2 liver cancer cell line are primarily a consequence of cell cycle arrest in the G2/M phase. Furthermore, the process of encapsulating glutathione in four-layer microcapsules composed of furcellaran and chitosan was successfully achieved using the layer-by-layer (LbL) technique, which protected the compound from degradation caused by environmental factors and gastrointestinal enzymes [4]. In vivo studies have shown that oral supplementation of encapsulated glutathione leads to increased serum concentrations, indicating the potential usefulness of this form in correcting deficiencies. The observed increases in antioxidant enzyme activity and modulation of gene expression, particularly those encoding glutathione reductase, suggest improved oxidative–antioxidant balance in rats receiving a diet enriched with microcapsules. Importantly, no adverse effects on liver or kidney function were noted, suggesting that oral administration of glutathione in furcellaran–chitosan microcapsules is safe. These findings highlight the potential of FUR/CHIT microcapsules as glutathione delivery systems. In the present study, a model food system was designed to further investigate their behaviour under in vitro digestion conditions.

### 2.1. Ingredient Composition and Microstructural Characteristics of the Glutathione-Enriched Model Product

A model snack bar intended for physically active individuals was developed, taking into account the nutritional requirements associated with peri-exercise consumption. The formulation was designed to provide an optimal energy source and to deliver naturally occurring compounds with the potential to mitigate exercise-induced oxidative stress. Gluten-free buckwheat and oat flakes formed the structural base of the matrix, ensuring the product was suitable for individuals following a gluten-free diet. The carbohydrate profile was shaped by incorporating ingredients with different glycaemic loads, buckwheat, oat flakes and pecan nuts (low GL), together with dried dates (medium GL), to enable both rapid and sustained carbohydrate release, thereby helping to maintain muscle performance during exercise.

To reduce the risk of gastrointestinal discomfort and avoid slowing gastric emptying, the fat content of the snack was kept intentionally low, with pecan nuts included only in small amounts to provide plant-derived lipids and naturally occurring antioxidants that support post-exercise recovery. Additional intrinsic sugars, fruit solids and phenolic compounds were supplied by dried dates, honey, freeze-dried raspberries and cherry juice, collectively resulting in a cereal- and fruit-based bar with a modest contribution of plant proteins from the raw materials. All five snack variants shared the same base formulation, differing only in the form (free or microencapsulated) and the method (internal incorporation or surface electrospraying) of glutathione addition.

**Figure 1:**
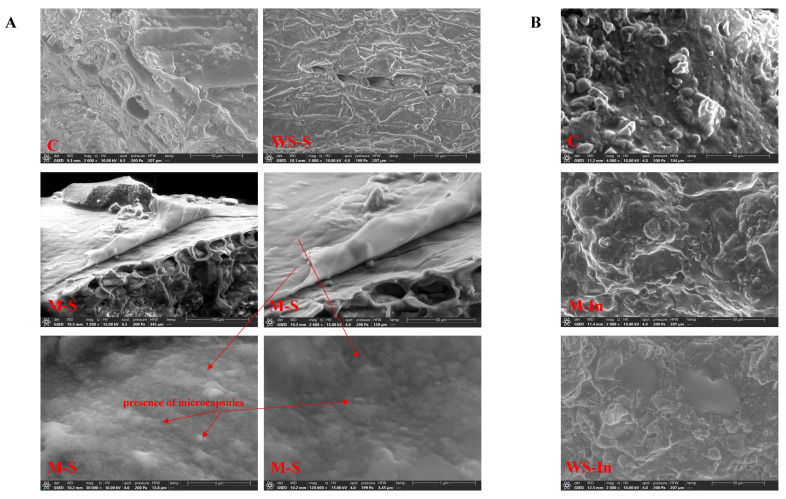
SEM images (**A**) of the surface of snacks, (**B**) of the cross-section of snacks. C, control version without added glutathione; M-S, microencapsulated glutathione applied to the surface via electrospraying; M-In, a version with microencapsulated glutathione added inside the product; WS-S, a snack containing glutathione in the form of an aqueous solution applied to the surface via electrospraying; WS-In, a variant in which an aqueous solution of glutathione was added inside the product. Microcapsules in the microstructure are indicated by red arrows.

The microstructure of the snack variants was assessed using scanning electron microscopy (Figure 1).

The surface of the control sample (C) and the variant with free glutathione solution applied by spraying (WS-S) exhibited similar morphology, although the WS-S sample presented crystal-like structures attributable to undissolved glutathione. In contrast, the sample with microencapsulated glutathione sprayed onto the surface (M-S) displayed a distinct additional layer, indicating the deposition of microcapsules. Higher magnification revealed agglomerates of both larger and smaller capsules. Cross-section images (Figure 1B) showed no visible structural differences between samples with glutathione added inside the matrix (M-In, WS-In) and the control product, which is consistent with the heterogeneous nature of the food matrix composed of flakes, fruit particles and nuts. Nonetheless, SEM imaging confirmed successful surface deposition of microcapsules in the M-S variant.

### 2.2. Bioaccessibility of Glutathione in Digests of the Model Product

The oral administration of bioactive compounds encounters multiple challenges, including degradation by proteolytic enzymes, significant pH variations, low solubility and restricted epithelial permeability. Encapsulation has been utilised as a strategy to mitigate these obstacles and improve absorption following oral intake [7].

**Figure 2:**
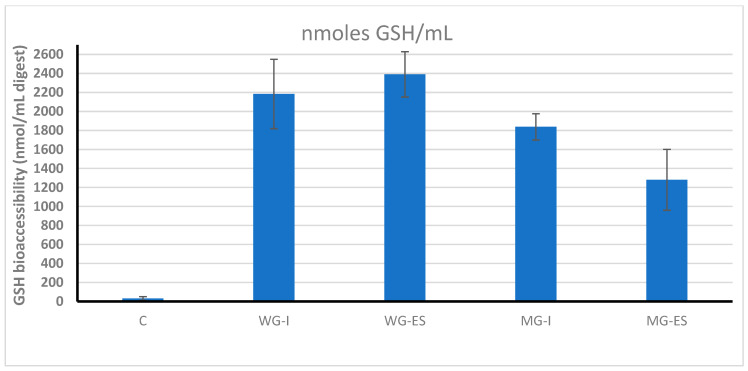
Glutathione content (GSH bioaccessibility (nmol/mL digest)) in the digestion products of a model product enriched with glutathione by various methods. Values are expressed as mean ± SD. Different lettering indicate significant differences (*p* < 0.05). C, control version without added glutathione; MG-ES, microencapsulated glutathione applied to the surface via electrospraying; MG-I a version with microencapsulated glutathione added inside the product; WG-ES, a snack containing glutathione in the form of an aqueous solution applied to the surface via electrospraying; WG-I, a variant in which an aqueous solution of glutathione was added inside the product.

In controlled drug delivery systems, the release of active compounds often relies on a mechanism known as polymer network swelling. When the system is introduced into an aqueous environment, such as bodily fluids, the polymer matrix absorbs water, causing it to expand. This swelling widens the pores within the polymer structure, creating pathways that allow the active drug to diffuse slowly into the surrounding environment until it reaches thermodynamic equilibrium. This controlled release mechanism is essential for maintaining steady drug levels over time, enhancing therapeutic efficacy and minimising side effects [8]. The degree of glutathione release, determined based on its content in the digestion products of the model product, is presented in Figure 2. Both variants of snacks with microencapsulated glutathione and control samples as well as the glutathione solution were analysed, enabling assessment of microencapsulation efficiency compared to other forms. It should be noted that, due to the encapsulation efficiency of approximately 70% reported for the applied system, a fraction of glutathione in the microcapsule-containing samples was present in a non-encapsulated form. Therefore, the GSH levels determined in the digesta reflect the combined contribution of both encapsulated and non-encapsulated fractions, rather than a purely controlled release from the microcapsules.

The obtained results indicate significant differences in the release behaviour of glutathione depending on the form in which it was added to the model food product. In the digestion products of the control sample, to which glutathione was not added, its natural occurrence was observed at a level of approximately 32 ± 7 nmol/mL, which suggests that the ingredients used to produce the snack, such as dates or freeze-dried strawberries, contain certain amounts of this compound [9]. It should be noted that the glutathione content reported in this study reflects the total amount detected in the digesta and was not corrected for the baseline level originating from the raw materials. However, the contribution of endogenous glutathione was negligible compared to the concentrations observed in enriched samples (over 1200–2300 nmol/mL) and therefore did not influence the comparative evaluation of different delivery systems. Significantly higher glutathione concentrations were observed in samples enriched with this compound, with the highest values obtained for the digestion products of snacks with the addition of the aqueous glutathione solution (2390 and 2180 nmol/mL), and the lowest values for the digestion products of snacks with glutathione capsules (1280 and 1840 nmol/mL). The lower glutathione concentration in the case of microcapsules may be associated with limited release of the encapsulated fraction of glutathione from the microcapsule matrix during digestion. During the digestion process, microcapsules may not completely disintegrate, as a result of which some glutathione remains enclosed in their matrix, and consequently, lower amounts are detected in the digesta. In contrast, glutathione added in the form of an aqueous solution is immediately available in the digestive environment, which explains the higher concentrations observed in these samples. However, this immediate availability does not allow conclusions regarding its physiological relevance, as the compound may be subject to faster degradation or be less efficiently absorbed by the body. Similar results were obtained in the research by Tavano, Muzzalupo [7], by evaluating the behaviour of niosomal preparations containing gallic acid and curcumin during in vitro digestion. The researchers found that the cumulative amounts of gallic acid and curcumin released from niosomal preparations in the gastrointestinal tract were lower compared to the corresponding solutions of free substances. According to Dias, Ferreira [6], microencapsulation of bioactive ingredients can significantly change their release and absorption profile. The authors emphasised that microencapsulation not only protects bioactive compounds from degradation but also enables gradual release in optimal digestive conditions, which may influence their release behaviour under digestive conditions. Moreover, the same authors highlighted that the encapsulation technique and the shell material are crucial for the bioaccessibility of the active substance. Therefore, it is possible that the developed innovative microcapsules were not completely soluble in in vitro digestion conditions, which could limit their diffusion into the enzymatic environment and reduce the determined bioaccessibility. These results indicate that the form of glutathione incorporation influences its release behaviour during digestion. However, it should be emphasised that the present study evaluates bioaccessibility in the digesta rather than actual absorption. Therefore, no conclusions can be drawn regarding the physiological relevance of the observed differences. Thus, further studies should be conducted, including assessment of the actual absorption of glutathione in vivo and analysis of microcapsule stability in different sections of the gastrointestinal tract.

### 2.3. Antioxidant Activity of Samples and Digests

**Figure 3:**
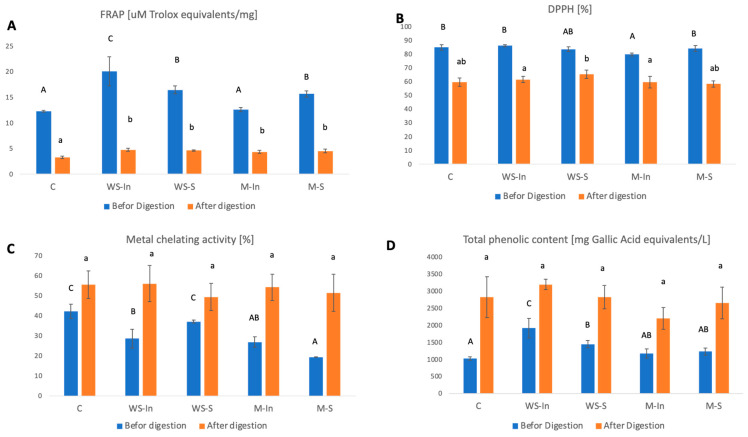
Antioxidant properties and phenolic compound content in digestion products of model foods; (**A**) FRAP; (**B**) DPPH; (**C**) Metal chelation; (**D**) Total phenolic content.Values are expressed as mean ± SD. Different lettering indicate significant differences (*p* < 0.05). Uppercase letters indicate statistical differences between groups before digestion. Lowercase letters indicate statistical differences between groups after digestion; C, control version without added glutathione; M-S, microencapsulated glutathione applied to the surface via electrospraying; M-In, a version with microencapsulated glutathione added inside the product; WS-S, a snack containing glutathione in the form of an aqueous solution applied to the surface via electrospraying; WS-In, variant in which an aqueous solution of glutathione was added inside the product.

As shown in Figure 3, after digestion, the FRAP and DPPH values of digests decreased while metal chelating activity values increased.

Before digestion, the highest reducing capacity (FRAP) was observed in samples enriched with glutathione in aqueous form, suggesting improved antioxidant activity. However, after digestion, no consistent or statistically significant differences between enriched samples and the control were observed, indicating that the form of glutathione incorporation did not significantly influence antioxidant reactivity under simulated gastrointestinal conditions. Similarly, all samples exhibited high DPPH radical scavenging activity before digestion, which decreased after digestion, with no clear effect of glutathione addition. This suggests that antioxidant activity in the digesta was primarily driven by other matrix components rather than glutathione.

The lack of observable differences may be explained by several factors. Glutathione does not exhibit strong activity in conventional assays such as DPPH and FRAP, which are primarily based on electron transfer mechanisms [10], and its antioxidant action is largely associated with intracellular redox systems rather than direct radical scavenging [11]. Moreover, its contribution may be masked by phenolic compounds and digestion-derived peptides present in the food matrix, which typically exhibit stronger responses in such assays [12]. Additionally, gastrointestinal conditions may lead to structural modification, oxidation, or interactions of glutathione with other components, reducing its detectability in in vitro assays [13]. In contrast, metal chelating activity increased after digestion in all samples, which is likely related to the release of phenolic compounds from the food matrix, known for their strong metal-binding properties. These findings indicate that conventional antioxidant assays may not be suitable to assess the functional performance of glutathione delivery systems in complex food matrices under simulated gastrointestinal conditions.

The total phenolic compounds in all samples before the in vitro digestion process was high and ranged from 1917 mg gallic acid equivalents/L acid (WS-In) to 1028 mg gallic acid equivalents/L (C). As a result of digestion, an increase in the content of phenolic compounds was observed in all samples (from 2200 mg GAE/L for sample M-In to 3190 mg GAE/L for sample WS-In). Statistically significantly higher content of phenolic compounds before digestion in the sample to which glutathione was added in the form of an aqueous solution (WS-In) was probably due to the easier release of these compounds from the food matrix and their potential stabilisation by glutathione [14]. In other samples, in which glutathione was applied by spraying or in the form of microcapsules, this effect did not occur, which may be due to several factors. First of all, microencapsulation may have limited the direct interaction of glutathione with phenolic compounds, preventing their protection from oxidation or increased extraction. Secondly, in the case of spraying, it was possible that glutathione was unevenly distributed onto the snack surface, which could have reduced its effect on the phenolic compound extraction process. However, further studies should be conducted to verify these hypotheses.

The increase in phenolic compound content after in vitro digestion observed in all tested samples may result primarily from their release from the food matrix, as well as changes in their extractability under varying pH conditions during digestion. The disruption of the food structure and hydrolysis of macromolecules may facilitate the liberation of phenolic compounds previously bound to proteins, polysaccharides, or other matrix components [15].

According to Wojtunik-Kulesza, Oniszczuk [16], digestive processes can significantly affect the bioactivity and availability of phenolic compounds, which explains the observed changes in their content after simulated digestion. The lack of differences in phenolic compound content after digestion between the studied groups suggests that the addition of glutathione, regardless of its form, had no significant effect on their release from the food matrix. Although the presence of other bioactive compounds may influence the behaviour of phenolics during digestion [17], no such effect was observed under the conditions applied in this study.

### 2.4. Effect of Microcapsule Addition on Protein Digestibility in Model Product

#### 2.4.1. Protein Digestibility Indices in Digestion Products

**Figure 4:**
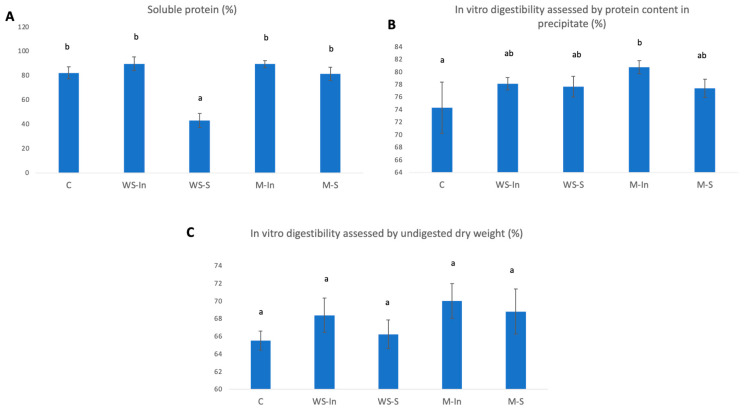
Protein digestibility indices in model product with added glutathione; (**A**) Soluble Protein; (**B**) Protein content in precipitate; (**C**) Undigested dry weight. Values are expressed as mean ± SD. Different lettering indicates significant differences (*p* < 0.05). C, control version without added glutathione; M-S, microencapsulated glutathione applied to the surface via electrospraying; M-In, a version with microencapsulated glutathione added inside the product; WS-S, a snack containing glutathione in the form of an aqueous solution applied to the surface via electrospraying; WS-In, variant in which an aqueous solution of glutathione was added inside the product.

Due to the diversity of origin and structure of food proteins, their presence in food matrices (such as cell walls, bonds with sugars, fibres, lipids), and the fact that they are simultaneously hydrolysed by a mixture of enzymes with different specificities—from narrow (trypsin) to broad (elastase, pepsin)—this process is characterised by complex kinetics. Various molecular structures appear in the intestinal lumen, from free amino acids to complex polypeptides, which are constantly modified as food moves through the digestive tract [18]. To assess the protein digestibility of the samples, we employed different analytical methods. Differences were found in the degree of digestibility of the products, expressed as the percentage of soluble proteins contained in the supernatants and protein content in the precipitate (Figure 4).

The WS-S sample, in which the aqueous glutathione solution was applied to the bar surface, showed a significantly lower level of soluble protein compared to the other variants (43%) (Figure 4A). However, this result should not be interpreted as reduced protein digestibility. The soluble protein assay primarily detects larger peptides and proteins present in the supernatant, while its sensitivity towards low-molecular-weight peptides and free amino acids is limited. Therefore, the lower soluble protein content observed in the WS-S sample is more likely related to a shift in the profile of digestion products rather than to a decrease in protein hydrolysis. It can be assumed that proteins in this system were more extensively degraded into smaller peptides and free amino acids, which are not efficiently captured by the protein assay. This interpretation is supported by the fact that no significant differences were observed in overall digestibility assessed by undigested dry residue, indicating that the total extent of digestion remained comparable across samples (Figure 4C).

When protein digestibility was evaluated based on protein content in the precipitate (Figure 4B), only minor differences were observed, with a significant increase detected exclusively in the M-In sample compared to the control, while no differences were found among glutathione-enriched variants. This suggests that the form of glutathione incorporation had only a limited effect on protein partitioning between soluble and insoluble fractions. Overall, the results indicate that the method of glutathione application influenced the distribution and molecular size of digestion products rather than the overall degree of protein digestion.

Shen, Apriani [19] analysed the effect of food matrix on the digestibility of microencapsulated tuna oil and showed that digestion efficiency can vary depending on the type of food system. The authors attributed these differences to interactions between microcapsules and food components, such as fibre and proteins, as well as to the microstructure of the product. In the present study, although all samples were based on the same food matrix and differed only in the method of glutathione incorporation (free form vs. microencapsulated), observable differences were still noted. These differences may be related to variations in the molecular forms of digestion products, including proteins, peptides, and free amino acids. However, further studies are required to confirm this hypothesis.

#### 2.4.2. Free Amino Acid Profile in Digestive Products

**Figure 5:**
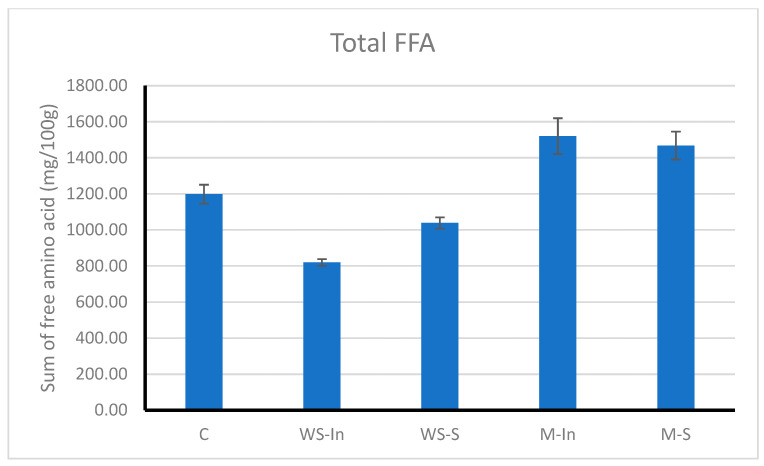
Total free amino acid content in the digests of model products. Values are expressed as mean ± SD. Different lettering indicates significant differences (*p* < 0.05): C, control version without added glutathione; M-S, microencapsulated glutathione applied to the surface via electrospraying; M-In, a version with microencapsulated glutathione added inside the product; WS-S, a snack containing glutathione in the form of an aqueous solution applied to the surface via electrospraying; and WS-In, a variant in which an aqueous solution of glutathione was added inside the product.

The total free amino acid content regarding the digestive products of the model products is shown in Figure 5.

The highest amounts of free amino acids were observed in the M-In (1520 mg/100 g) and M-S (1470 mg/100 g) samples. This may suggest that the presence of microencapsulated glutathione was associated with a higher proportion of low-molecular-weight digestion products. Microcapsules could provide a more gradual release of glutathione, which may influence its interactions within the food matrix during digestion. In contrast, free glutathione (WS-In and WS-S) may undergo faster transformations, including oxidation, which could influence its reactivity within the system. Additionally, components of microcapsules, such as proteins or polysaccharides, have been reported to create microenvironments that may affect enzymatic processes [20].

The lowest amount of free amino acids was found in the WS-In sample (820 mg/100 g), despite its relatively high value of soluble protein. This may indicate that proteins in this system were less extensively degraded to their final products, although still present in soluble forms detectable by the BCA method. Such an effect may be related to interactions between glutathione and proteins or enzymes, potentially influencing the accessibility of cleavage sites or enzyme activity. Mechanisms such as the formation or rearrangement of disulfide bonds, known to affect protein structure and digestibility [21], or interactions with proteolytic enzymes [22], have been reported in the literature. However, the molecular interactions responsible for the observed differences in amino acid profiles were not elucidated within the scope of the present study.

Overall, the obtained results suggest that both the form of glutathione and the method of its incorporation into the product may have influenced the course of protein digestion, particularly in terms of the nature of the resulting products. At the same time, no clear differences were observed in the overall extent of protein hydrolysis among the analysed samples.

It should be noted that the absence of a control consisting of empty FUR/CHIT microcapsules represents a limitation of the present study. Therefore, the effects observed in the M-In and M-S samples cannot be attributed exclusively to glutathione, as the polysaccharide shell itself may also influence digestion processes, for example, by affecting viscosity, enzyme interactions, or substrate accessibility. However, the lack of consistent trends across all analytical parameters suggests that the observed effects are not solely driven by the polysaccharide shell.

Further studies are needed to better disentangle the relative contributions of glutathione and the capsule matrix, for example, by applying alternative model systems or complementary analytical approaches that allow more precise assessment of their individual effects during digestion.

#### 2.4.3. Effect of Microcapsule Addition on Fat Digestibility in Model Product

**Figure 6:**
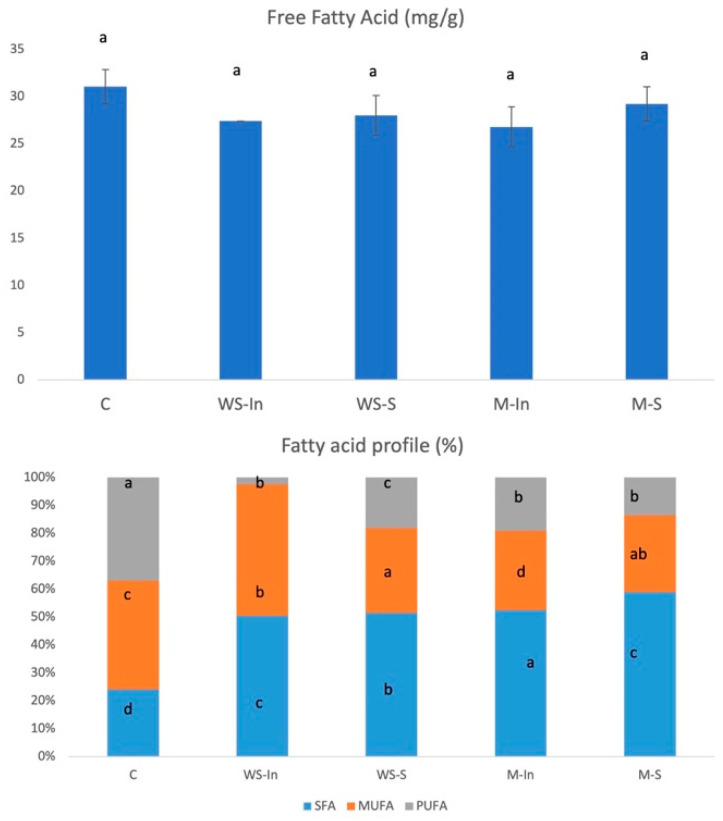
Free fatty acid content and fatty acid profile in the digest of model product with added glutathione. Values are expressed as mean ± SD. Different lettering indicate significant differences (*p* < 0.05). C, control version without added glutathione; M-S, microencapsulated glutathione applied to the surface via electrospraying; M-In, a version with microencapsulated glutathione added inside the product; WS-S, a snack containing glutathione in the form of an aqueous solution applied to the surface via electrospraying; WS-In, variant in which an aqueous solution of glutathione was added inside the product (WS-In).

In the current study, lipid digestion was also compared in the samples during simulated digestion processes (Figure 6).

In Figure 6, the content of free fatty acid released are shown for the samples measured using this solvent extraction method (31–27 mg/g free fatty acid). No statistically significant differences were found in this respect between the examined groups. This means that lipolytic enzymes degrade fats to a similar extent in all samples, regardless of the presence of free glutathione or microcapsules. At the same time, the analysis of the fatty acid profile showed significant differences in the ratio of saturated (SFA), monounsaturated (MUFA) and polyunsaturated fatty acids (PUFA) in the digestion products. Samples with added glutathione (both M-In, M-S, WS-In and WS-S) demonstrated significantly higher levels of saturated fatty acids (SFA) and lower levels of mono- (MUFA) and polyunsaturated (PUFA) fatty acids compared to the controls (C). The observed increase in the relative proportion of saturated fatty acids (SFA) in samples enriched with glutathione should not be interpreted as a conversion of unsaturated fatty acids into saturated ones, as such transformation is not expected under the applied conditions. Instead, these differences may be associated with the higher susceptibility of polyunsaturated fatty acids (PUFA) to oxidative degradation during in vitro digestion; however, this remains a hypothesis, as specific lipid oxidation products were not quantified in the present study. This is particularly relevant, as glutathione is widely recognised as a strong antioxidant that could potentially limit lipid oxidation processes. However, under the conditions of the applied model, no protective effect towards PUFA was observed, suggesting a limited or context-dependent effectiveness of its action in this system.

Under simulated gastrointestinal conditions, PUFA may undergo partial oxidation, leading to the formation of secondary products that are not detected as fatty acids in the analytical procedure [23]; however, this process was not directly assessed in the present study. This process may result in an apparent decrease in PUFA content, as these compounds are consumed during oxidation reactions [24]. Furthermore, lipid oxidation during digestion has been shown to occur independently of the overall extent of lipolysis [25], which is consistent with the present results, where no differences in total free fatty acid release were observed between samples. The lack of similar changes in the control sample suggests that the addition of glutathione or microcapsules may be associated with differences in the oxidative stability or analytical recovery of PUFA.

Taken together, the results indicate that glutathione, regardless of its form of application, did not influence the overall extent of lipid hydrolysis, while the observed changes in fatty acid profiles may be associated with oxidative transformations of PUFA occurring during digestion, although this mechanism was not directly confirmed.

## 3. Materials and Methods

### 3.1. Chemicals

L-Glutathione reduced (GSH) (CAS No. 70-18-8) was obtained from Sigma Aldrich (St. Louis, MO, USA). Furcellaran (FUR) (type 7000) was sourced from Est-Agar AS (Karla village, Estonia). The chemical composition of FUR (Mw = 2.951 × 10^5^) consisted of 79.61% carbohydrates, 1.18% protein and 0.24% fat, according to supplier specifications. Furcellaran was used as received from the supplier, and its degree of sulfation was not determined in the present study.

Chitosan (CHIT) (CAS No. 9012-76-4) was acquired from POL-AURA (Zabrze, Poland). The reported molecular weight (~890,000 Da), degree of deacetylation (≥90%), viscosity (100–300 cP) and particle size (≤100 mesh) were based on supplier specifications. The analytical methods used for determining these parameters were not provided by the manufacturer; therefore, these values should be considered approximate.

The polymers used in this study were consistent with those applied in our previous studies [26,27,28,29], in which furcellaran- and chitosan-based systems were extensively used in the development of biopolymer matrices and delivery systems. Although a full physicochemical characterisation of all parameters was not performed within the scope of the present work, the materials have been previously applied in comparable systems, supporting the consistency and reproducibility of the experimental design.

Sodium hydroxide (>99% purity) was also purchased from Sigma Aldrich (St. Louis, MO, USA).

### 3.2. Obtaining Double-Layer Furcellaran/Chitosan Microcapsules Encapsulating Glutathione

**Figure 7:**
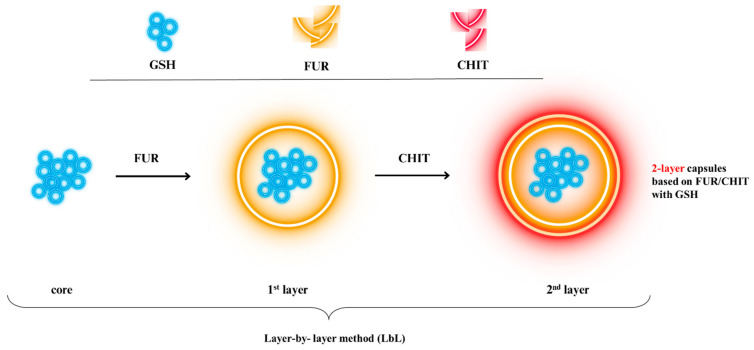
Procedure of obtaining 2-layer capsules based on FUR/CHIT with GSH.

The preparation and detailed characterisation of double-layer glutathione capsules have been reported previously [4,5], and the microcapsules used in this study were derived from the same production batch. The scheme of obtaining capsules was presented in Figure 7.

### 3.3. Design and Production of Model Product with Microencapsulated Glutathione Supporting Athletes’ Performance

A model snack was formulated from oat flakes (16.7%), dried dates (12.6%), buckwheat flakes (21.0%), pecan nuts (4.2%), freeze-dried raspberries (2.5%), honey (16.7%) and cherry juice (7.3%). The basic nutritional composition of the control variant was determined according to AOAC standard methods to characterise the baseline macronutrient profile of the model product. The control sample contained 10.4 ± 0.4% moisture, 7.4 ± 0.4% protein, 6.0 ± 0.3% fat and 0.65 ± 0.18% ash (wet basis). Since glutathione was added in relatively small amounts and did not replace any of the main ingredients, its incorporation did not significantly affect the overall macronutrient composition of the other variants.

**Figure 8:**
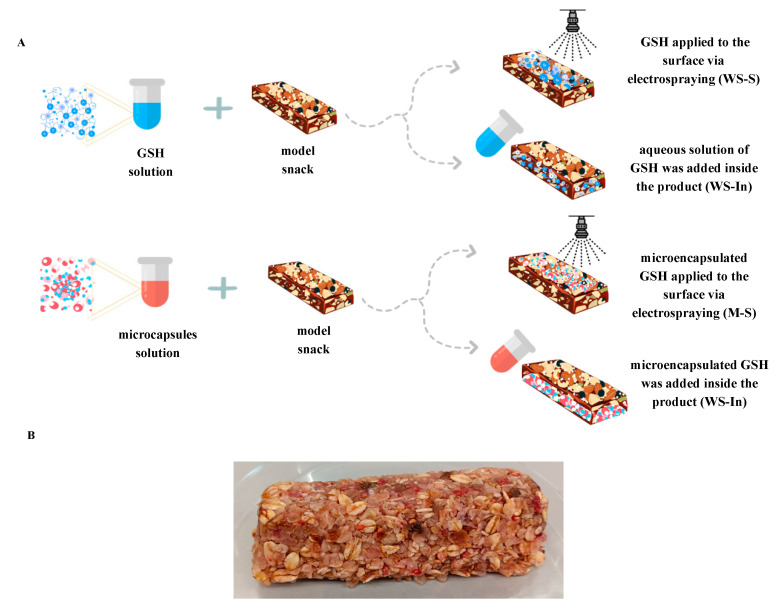
(**A**) The procedure for obtaining different types of snacks; (**B**) the appearance of the snack.

Water or an aqueous glutathione solution containing 500 mg of the compound, or a solution of glutathione microcapsules with an equivalent glutathione content, was added to obtain the required consistency. All components were manually mixed and shaped into 85 g bars. The products were stored at 4 °C until further analysis. Five snack variants were prepared for further research: a control version without added glutathione (C), a variant with microencapsulated glutathione applied to the surface via electrospraying (M-S), a version with microencapsulated glutathione added inside the product (M-In), a snack containing glutathione in the form of an aqueous solution applied to the surface via electrospraying (WS-S) and a variant in which an aqueous solution of glutathione was added inside the product (WS-In). Both the aqueous glutathione solution and glutathione microcapsules were applied to the snack surface using the electrospraying technique. For this purpose, the Fluidnatek FL-10 system (Bioinicia, Valencia, Spain) equipped with a scanning emitter and a vertical electrospray configuration was used. To ensure the appropriate spray angle, the industrial Euspray M-C2 (Barcelona, Spain) spray nozzle with a spray angle of 95° was applied. The spraying process was performed at a maximum flow rate of 6000 mL/h and a voltage drop of 20 kV between the emitter and the collector. GSH losses during the electrospraying process were determined to be approximately 10% based on a comparison of its content in the starting solution and the final product. The internal incorporation approach was also used to minimise potential losses associated with electrospraying and to provide a reference for evaluating its impact. All bars were prepared in three series, and five replicates from each series were taken for analysis. The preparation scheme of snacks for the study and the appearance of snacks are presented in Figure 8. No visible differences in appearance were observed between the control and fortified variants.

### 3.4. Microstructural Characterisation of Glutathione-Enriched Model Product via Scanning Electron Microscopy

The morphology and microstructure of the model snacks were characterised using the Quattro ESEM microscope (Thermo Fisher Scientific, Waltham, MA, USA) equipped with FEG (field emission gun, ETD, Everhart-Thornley detector). SEM images of sample cross-sections and surface were performed in LVD (low vacuum detector) mode at 200 Pa, magnifications ranging from 1200 to 120,000 times and with the accelerating voltage of 15 kV. The Thermo Scientific ChemiSEM Technology was used to explore the elemental composition of chosen samples imaged in ColorSEM mode.

### 3.5. In Vitro Digestion of a Model Product

Samples from an experimental and a control group underwent digestion using the static in vitro gastrointestinal simulation method (INFOGEST), as described by Brodkorb, Egger [30]. Before initiating digestion, all snack bars were mechanically pre-processed to simulate mastication: each 5 g portion was cut into small pieces and homogenised in a laboratory blender to obtain a uniform particle size suitable for the oral phase of the protocol. Prior to the digestion procedure, the enzymatic activity of the implemented digestive enzymes was evaluated according to the established protocol. Both samples and stock solutions were preheated to 37 °C, and all digestion steps were conducted at this temperature. In the oral phase, 5 mL of simulated saliva was introduced into a beaker containing 5 g of either the control or experimental sample. The mixture was stirred for two minutes to replicate the mechanical agitation occurring in the human mouth, ensuring an amylase activity of 75 U/mL in the final oral solution. During the gastric phase, simulated gastric fluids were incorporated into the oral phase mixture in a 1:1 volume ratio, followed by an immediate pH adjustment to 3.0. The final gastric mixture exhibited a pepsin activity of 2000 U/mL. Protein digestion within the stomach environment proceeded at pH 3.0 for two hours. In the small intestine phase, 20 mL of simulated intestinal fluids was added to the gastric phase mixture, and the pH was adjusted to 7.0. The final intestinal digestion solution contained bile salts at a concentration of 10 mM, with pancreatin activity reaching 100 U/mL. The mixture was continuously stirred for two hours to simulate intestinal conditions. Immediately following digestion, all samples were rapidly frozen using liquid nitrogen. After thawing, they were centrifuged at 13,000× *g* for 15 min at 4 °C to separate the soluble and insoluble fractions, with the supernatant and pellet collected separately.

#### 3.5.1. Analysis of Glutathione Bioaccessibility in the Digestion Products of a Model Product

The Glutathione Assay Kit (Catalogue No. CS0260, Sigma-Aldrich, Waltham, MA, USA) was used according to the manufacturer’s user manual. A 200 µL aliquot of the digest was taken, and 200 µL of 5% 5-Sulfosalicylic Acid Solution was added. Each sample was then vortexed vigorously and left for 10 min at 2–8 °C. Next, the samples were centrifuged at 10,000× *g* for 10 min and used for glutathione analysis.

#### 3.5.2. Influence of Digestion on Antioxidant Properties and Phenolic Compounds Content in Model Products

Before starting the digestion process of the model product, the extraction of antioxidant compounds was carried out according to the method described by Pérez-Jiménez and Saura-Calixto [31]. All analyses were performed not only for the model product before digestion, but also for the digestion products obtained after the simulated in vitro digestion process. After digestion, the samples were analysed directly, without prior dilution. The antioxidant properties were analysed by three methods: the iron ion reduction ability (FRAP) test, the assessment of the DPPH free radical scavenging activity and the measurement of the metal chelating ability. The FRAP test was used to assess the ability of the samples to reduce Fe^3+^ ions according to the method developed by Benzie and Strain [32]. The FRAP solution was prepared by mixing 300 mM acetate buffer (pH 3.6), 20 mM FeCl_3_·3H_2_O and 20 mM 2,4,6-tris(2-pyridyl)-s-triazine (TPTZ) solution in 40 mM HCl. The reagents were then mixed with the sample in a volume ratio of 0.4:3.6 and incubated in the dark for 10 min at 37 °C. After this time, the absorbance at 593 nm was measured using a Helios Gamma UV-1601 spectrophotometer (Thermo Fisher Scientific, Waltham, MA, USA). The DPPH radical scavenging activity was determined by mixing the sample with a DPPH solution in ethanol (0.1 mM) at a ratio of 0.2:2.8. The mixture was incubated for 10 min at room temperature, protected from light. After incubation, the absorbance was measured at 517 nm using a Helios Gamma UV-1601 spectrophotometer (Thermo Fisher Scientific, Waltham, MA, USA). The iron(II) chelating capacity was analysed according to the method described by Xie, Huang [33]. The absorbance was measured at 562 nm using Helios Gamma UV-1601 spectrophotometer (Thermo Fisher Scientific, Waltham, MA, USA). The content of phenolic compounds in hydrolysates was determined using the method proposed by Zaky, Chen [34] using gallic acid as a standard. Results are expressed as mg of gallic acid per millilitre.

#### 3.5.3. Effect of Microcapsule Addition on Protein Digestibility in Model Product

##### Solubilised Protein Determination Post-Digestion

The soluble protein content of the samples following in vitro gastrointestinal digestion was assessed using the supernatant from the digestates, blanks and controls according to the method described by Cutroneo, Prandi [35]. For all samples, total protein content was determined using the BCA assay (Cat. No.: 23225, Thermo Fisher Scientific) on 1 mL of supernatant. The soluble protein content, expressed as a percentage, was then calculated by comparing the actual protein content—after subtracting the blank—of the supernatants to the estimated total protein content of the initial sample before digestion.

##### In Vitro Digestibility Assessed by Undigested Dry Weight

The in vitro digestibility was assessed according to the method described by Wen, Zhou [36]. After digestion, the resulting mixture was centrifuged at 10,000× *g* for 20 min at 4 °C, and the supernatant was discarded. The precipitate was then dried at 50 °C until it reached a constant weight. The degree of digestibility was assessed following Equation (1) by determining the weight of the dried insoluble protein fraction remaining after in vitro digestion (W_i_) and comparing it with the total weight of the dried protein powder prior to digestion (W_t_).

Digestibility = (1 − W_i_/W_t_) × 100% (1)

##### In Vitro Digestibility Assessed by Protein Content in Precipitates

The in vitro digestibility was assessed according to the method described by L. Li et al. (2017) [37]. Following the digestion procedures, the resulting mixtures were centrifuged at 10,000× *g* for 20 min at 4 °C. The protein content in the precipitates was then measured using the BCA method with a commercial kit assay (Cat. No.: 23225, Thermo Fisher Scientific). The degree of digestibility was calculated

Digestibility (%) = (W0 − W1)/W0 × 100%(2)

where W1 is the protein content (g) in the precipitate after digestion, and W0 is the protein content (g) in the untreated product before digestion.

##### Free Amino Acid Content in Digest

For the free amino acid analysis, the 1 mL of digest was a lyophilisate. Then the samples (100 mg dissolved in 10 mL of 0.1 M HCl) were sonicated in an ultrasonic bath (Polsonic, Palczyński, Warsaw, Poland) for 20 min. The mixture was then centrifuged at 3000× *g* for 10 min at 4 °C.

For derivatisation, 10 mL of the clarified extract was mixed with 70 μL of borate buffer (pH 8.2–9.0) and 20 μL of 6-aminoquinolyl-N-hydroxysuccinimidyl carbamate (AQC) dissolved in acetonitrile (3 mg/mL). Standards were prepared using the same procedure. The samples were appropriately diluted before analysis and derivatised by mixing 10 μL of each sample with 70 μL of borate buffer (pH 8.2–9.0) and 20 μL of the AQC reagent in acetonitrile.

Chromatographic separation was conducted on a Dionex Ultimate 3000 HPLC system (Thermo Scientific, Waltham, MA, USA) equipped with an LPG-3400 SD four-channel gradient pump, a WPS-3000 TSL autosampler and an FLD-3400RS four-channel fluorescence detector. The analyses were performed on a Nova-Pak C18 column (4 μm, 150 × 3.9 mm; Waters, Milford, MA, USA). The mobile phases consisted of (A) an acetate–phosphate buffer and (B) a 60:40 (*v*/*v*) acetonitrile–water mixture. The column temperature was maintained at 37 °C. Fluorescence detection was carried out at an excitation wavelength of 250 nm and an emission wavelength of 395 nm. Quantification was based on a one-point calibration using analytical standards (50 pmol for each amino acid).

#### 3.5.4. Effect of Microcapsule Addition on Fat Digestibility in Model Product

##### Free Fatty Acid Content in Digest

To determine the fraction of free fatty acid released from the samples during in vitro digestion, a modified titration method was employed according to Zhou, Hu [38]. Specifically, 2 mL of the digestion sample was placed in a test tube, followed by the addition of 5 mL of chloroform (2:1 *v*/*v*) for extraction. After centrifuging at 4000× *g* for three minutes, the lower phase was carefully transferred to a 300 mL flask. To this, 100 mL of ether/ethanol (1:1) and two to three drops of phenolphthalein were added. The mixture was then titrated with 0.01 M NaOH until the colour changed from colourless to light red, and the volume of used NaOH was recorded. The amount of free fatty acids released per gram of the sample was determined by considering the volume of NaOH used during titration, the concentration of the NaOH solution and the molecular weight of the free fatty acids.

##### Free Fatty Acid Profiles in Digest

For the free fatty acid analysis, the 1 mL of digest was a lyophilisate. Fat was extracted from samples using the Soxhlet method. Fatty acid methyl esters underwent analysis via the TRACE 1300 GC (Thermo Scientific, Waltham, MA, USA) equipped with a FID detector. The separation was carried out on a Zebron ZB-FAME 60 m × 0.25 mm × 0.20 mm column (Phenomenex, Torrance, CA, USA). The designated carrier gas was helium, with a 5 mL/min flow. The split flow was adjusted to 10 mL/min. The temperatures applied in the trial were 240 °C (detector) and 220 °C (feeder). There was an increase in column temperature by 3 °C/min, from 140 °C, with a 1 min hold, to 220 °C, the hold being 5 min. Fatty acid methyl ester identification was performed by comparing the retention times with the Supelco 37 FAME Mix standards (Sigma-Aldrich, Waltham, MA, USA).

### 3.6. Statistical Analysis

Statistical analysis was performed using Statistica 13.3 (TIBCO Software Inc., USA). Three independent production series of the snack were prepared. Within each series, five samples were analysed, and each measurement was performed in two technical replicates.

The Shapiro–Wilk test was used to verify the normality of data distribution, and the homogeneity of variances was assessed using Levene’s test.

The results were analysed using one-way analysis of variance (ANOVA), followed by Tukey’s post hoc test. Tukey’s test inherently accounts for multiple comparisons by controlling the family-wise error rate. Differences between mean values were considered statistically significant at *p* < 0.05.

## 4. Conclusions

This study evaluated the behaviour of glutathione (GSH), applied in free and microencapsulated forms, in a model food system under simulated in vitro digestion conditions. The results showed that the form of glutathione incorporation influenced its release into the digesta, with higher concentrations observed for free GSH compared to the microencapsulated form. These differences reflect variations in release behaviour during digestion rather than differences in overall bioaccessibility. No consistent effect of glutathione addition or microencapsulation was observed on antioxidant activity measured in the digesta, indicating that the results were primarily driven by other components of the food matrix. The applied analytical approaches to protein digestion indicated differences in the distribution and molecular size of digestion products, while no clear differences were observed in the overall extent of protein hydrolysis between samples. Similarly, total fat digestibility did not differ between variants, although changes in fatty acid profiles were observed, which may be related to transformations occurring under in vitro digestion conditions. Overall, the results demonstrate that the form and method of glutathione incorporation may influence its behaviour within a complex food matrix during digestion. However, due to the limitations of the static in vitro model, these findings should be interpreted with caution, and further studies are required to assess their physiological relevance.

## Data Availability

The raw data supporting the conclusions of this article will be made available by the authors on request.
